# Differences in electroencephalographic non-rapid-eye movement sleep slow-wave characteristics between young and old mice

**DOI:** 10.1038/srep43656

**Published:** 2017-03-03

**Authors:** Maria Panagiotou, Vladyslav V. Vyazovskiy, Johanna H. Meijer, Tom Deboer

**Affiliations:** 1 Laboratory for Neurophysiology, Department of Molecular Cell Biology, Leiden University Medical Centre, 2333 ZC Leiden, The Netherlands; 2 Department of Physiology, Anatomy and Genetics, University of Oxford, OX1 3PT Oxford, UK

## Abstract

Changes in sleep pattern are typical for the normal aging process. However, aged mice show an increase in the amount of sleep, whereas humans show a decrease when aging. Mice are considered an important model in aging studies, and this divergence warrants further investigation. Recently, insights into the network dynamics of cortical activity during sleep were obtained by investigating characteristics of individual electroencephalogram (EEG) slow waves in young and elderly humans. In this study, we investigated, for the first time, the parameters of EEG slow waves, including their incidence, amplitude, duration and slopes, in young (6 months) and older (18–24 months) C57BL/6J mice during undisturbed 24 h, and after a 6-h sleep deprivation (SD). As expected, older mice slept more but, in contrast to humans, absolute NREM sleep EEG slow-wave activity (SWA, spectral power density between 0.5–4 Hz) was higher in the older mice, as compared to the young controls. Furthermore, slow waves in the older mice were characterized by increased amplitude, steeper slopes and fewer multipeak waves, indicating increased synchronization of cortical neurons in aging, opposite to what was found in humans. Our results suggest that older mice, in contrast to elderly humans, live under a high sleep pressure.

Aging has been associated with numerous and diverse changes in sleep. In humans, these include increased sleep fragmentation, decreased total sleep time, sleep efficiency and slow-wave-sleep (SWS), and attenuated electroencephalogram (EEG) slow-wave activity (SWA, EEG power density between 0.75–4 Hz) in non-rapid eye movement (NREM) sleep^1^,^2^,^3^,^4^. In parallel with SWA, the amplitude, slope and density of the NREM sleep slow waves was decreased in middle aged (41–60 y.o.) and older humans (50–70 y.o.)^5^,^6^. In mice, aging impacts the amplitude and timing of circadian rhythms as well as sleep quality^7^,^8^,^9^,^10^,^11^,^12^ and waking performance^13^,^14^,^15^.Several studies showed that the time spent asleep over 24 hours increased with age in mice, particularly during the dark period^7^,^8^,^9^,^10^,^11^,^12^. Since aged mice show increased sleep fragmentation and increased sleep propensity in their habitual waking period during the night as well as less pronounced circadian rhythms of sleep and wakefulness^7^,^11^, they have been considered a good model to investigate age-dependent changes in sleep physiology. In humans, changes in NREM sleep SWA and slow-wave characteristics were studied thoroughly and were found to be consistent with the decrease in the amount of sleep seen in aging^1^,^2^,^3^,^4^,^5^,^6^. However, in mice the effects of age on slow-wave parameters, that could provide insight into cortical network properties, have not been studied, and it remains unknown whether EEG changes occur in parallel with global sleep-wake alterations.

Sleep is considered to be mainly regulated by two processes^16^,^17^. The timing of sleep is regulated by the circadian clock which in mammals is located in the suprachiasmatic nucleus (SCN) of the hypothalamus. The depth of sleep is homeostatically regulated, as reflected in progressively increasing sleep propensity during wakefulness and its dissipation during sleep. Prolonged waking is compensated by deeper and sometimes longer subsequent sleep^16^. In mammals, the homeostatic sleep process is thought to be reflected in the NREM sleep EEG SWA^16^. The overall level of EEG SWA is determined mostly by the incidence, as well as amplitude of individual EEG slow waves, which show distinct changes across the sleep period^18^,^19^,^20^. In addition, slow-wave slopes and the number of peaks within a single wave (multipeak waves) are thought to reflect network synchronization^18^,^19^,^20^,^21^. During early sleep, when SWA is high, there are more slow waves with large amplitude, their slopes are steep, and multipeak waves are rare^18^,^19^,^20^. As sleep progresses, SWA diminishes, reaching lowest levels at the end of the main sleep period^16^,^22^. During that period there are fewer large-amplitude slow waves, their slopes are less steep, and multipeak waves are more frequent^18^,^19^,^20^. Sleep deprivation (SD) experiments have shown that SWA in NREM sleep increases as a function of prior waking duration and decreases again during subsequent recovery sleep^16^. The incidence and morphology of the slow waves were shown to follow an analogous pattern with increased incidence, amplitude and slopes and decreased multipeak waves^18^,^19^,^20^.

Investigating the parameters of individual slow waves, such as the amplitude, duration and slopes, provides important insights into the functioning of the underlying neuronal network. It has been established, that EEG slow-wave fluctuations reflect synchronized activity of large cortical populations near the recording electrode, as well as activity originating from distant sources, influencing the activity patterns among neurons, located near the electrode^23^. Previous studies, where EEG or local field potentials were combined with cortical neuronal activity recordings, revealed that sleep slow waves are a reflection of near-synchronous transitions, between up (active) and down (silent) state in large populations of neurons, as well as, that steeper slow-wave slopes are associated with tighter synchrony at the transitions between activity and silence^18^,^24^,^25^,^26^,^27^,^28^. The observations from the aforementioned research studies provide the opportunity to study the network mechanisms, underlying age-dependent changes in cortical oscillations, by recording the NREM sleep EEG in the aging mouse model.

In this study, we investigated for the first time, the incidence and characteristics of NREM sleep slow waves, including their amplitude, duration, number of peaks within a single wave and slopes in the aging mouse model to define the cortical network characteristics. We conclude that the underlying cortical network likely shows increased synchronization in older mice (18–24 months old) compared to young controls (6 months old), resembling the effects of an increase in sleep pressure.

## Materials and Methods

### Animals

Young (6 months old; n = 9) and older (18–24 months old; n = 24) male C57BL/6JOlaHsd mice (Harlan, Horst, the Netherlands) were used for this study. All mice were housed under controlled conditions (12:12 h light:dark cycle; lights on at 09:00) with food and water *ad libitum* in a temperature controlled room (23–24 °C). The animals were individually housed in cages with a running wheel three months prior to the experiment for cage enrichment and increased chances of healthy aged mice^29^. All animal experiments were approved by the Animal Experiments Ethical Committee of the Leiden University Medical Center (the Netherlands) and were carried out in accordance with the EU Directive 2010/63/EU on the protection of animals used for scientific purposes.

### Surgeries

When the animals reached the required age, they were operated under deep anesthesia (Ketamine 100 mg/kg; Xylazine 10 mg/kg; Atropine 1 mg/kg) and EEG recording screws and electromyogram (EMG) electrodes (Plastics One) were implanted as described previously^30^,^31^. One EEG electrode was placed over the right hemisphere (2 mm lateral to the midline of the skull, 2 mm posterior to bregma) above the somatosensory cortex, while the other was placed above the cerebellum (midline, 2 mm caudal to the lambda) as a reference. The wire branches of all electrodes were set in a plastic pedestal (Plastics One, Roanoke, VA) which was fixed to the skull with dental cement. The mice were left to recover for at least 7–10 days.

### EEG recordings

The EEG and EMG were recorded with a portable recording system (PS 1 system, Institute of Pharmacology and Toxicology, Zurich, Switzerland) as previously described^30^,^31^,^32^. Before each recording, a calibration signal (10 Hz sine wave 300 μV peak-to-peak) was recorded on the EEG and EMG channels. Both signals were amplified (amplification factor ~2,000), conditioned by analogue filters (high-pass filter −3 dB at 0.16 Hz) and sampled at 512 Hz. The signals were filtered through a digital finite impulse response filter (EEG low-pass filter at 30 Hz and EMG band-pass filter between 20 and 40 Hz) and stored with a resolution of 128 Hz (with 12 bit amplitude resolution). EEG power spectra were computed for 4-s epochs.

To record the EEG and EMG, animals were placed into experimental chambers and connected through a flexible cable and a counterbalanced swivel system to the recording setup. Conditions in the experimental chamber were similar to the home cage. Before starting the experiment, the animals were allowed to adjust to the experimental conditions for at least a week. Subsequently, a baseline (BL) day was recorded, starting at lights on. At the start of the second day, six hours of SD were conducted by gentle handling^30^,^33^. During that period, every time the animals appeared drowsy or the EEG exhibited slow waves, the animals were mildly disturbed by noise, movement of bedding or introducing new nesting material or food into the cage. EEG and EMG were recorded continuously during SD and, subsequently, for 18 hours to investigate sleep after SD.

### Data analysis and statistics

Three vigilance states (REM sleep, NREM sleep and Waking) were scored offline in 4 s epochs by visual inspection of the EEG and EMG signals as well as EEG power density in the slow-wave range, as described previously^30^,^31^. Epochs with artifacts were excluded from the subsequent data analysis of the power spectra, but vigilance states could always be determined. Corresponding light and dark mean values and 2-hourly values of vigilance states and EEG SWA in NREM sleep were analyzed. The distribution of waking, NREM sleep and REM sleep episode duration was analyzed according to previously published criteria^34^,^35^,^36^. Episodes of each vigilance state were partitioned into ten bins with exponential increased duration from 4 s to >1024 s. Spectral analysis was performed using a fast Fourier transform (FFT; 0.5–25 Hz, 1 Hz resolution). Wake, NREM and REM sleep EEG spectra were computed during the BL. Absolute values for the SWA were computed to increase the possibility to compare this with the routinely available absolute slow wave data in humans^2^,^5^,^19^,^37^,^38^. The decay rate in NREM sleep SWA after SD was calculated between the first and third hour, by calculating the proportion of the first 30 min-value after SD to the sixth 30 min-value (3^rd^ hour) after SD.

A one-way, two-way or three-way analysis of variance (ANOVA) with factors age, time of the day, day, Light-dark, duration/frequency bin, and amplitude levels, was applied to test the differences between the age groups and experimental conditions. When the ANOVA reached significance, paired and unpaired t-tests were applied when appropriate to determine the effects of SD or age. R-values were averaged after fisher-Z transformation.

### Detection and Analysis of Slow Waves

Detection of individual slow waves was performed on the EEG signal after band pass filtering (0.5–4 Hz, stopband edge frequencies 0.1–10 Hz) with MATLAB filtfilt function exploiting a Chebyshev Type II filter design (MATLAB, The Math Works, Inc., Natick, MA)^39^. As a first step, slow waves were detected both as positive and negative deflections of the EEG signal between two consecutive negative (or positive respectively) deflections relative to the zero-line separated by at least 0.1 seconds. Subsequently, for each animal, we calculated independently integrated EEG amplitude in a spindle frequency range (band pass filter: 9–13 Hz, stopband edge frequencies 7–15 Hz, Chebyshev Type II filter design, MATLAB, The Math Works, Inc., Natick, MA), and computed slow-wave triggered average of this new time series both for positive and negative slow waves. As it is well known, population ‘down-states’ correspond to reduced faster EEG frequencies, while spindles are often associated with an up-state both in humans and animals^40^,^41^,^42^. The resulting two time series were then subjected to further analysis. For the final analyses, we selected for each individual animal either positive or negative EEG slow waves, which corresponded to a suppression of corresponding amplitude of EEG signal in a spindle-frequency range. This procedure ensured that all analyses were performed not on arbitrarily selected EEG negative waves, but on EEG potentials corresponding to reduced network activity. There is evidence, that the first segment of slow waves corresponds to the down phase of the slow oscillations, whereas the second segment corresponds to the up phase^39^,^40^,^43^,^44^. The peak-to-peak amplitude of the first and second segment of the wave, the slopes (mean first derivative of the first and second segment), and the number of peaks within a single wave were computed for each individual wave during NREM sleep. Multipeak waves were defined as waves with more than 1 peak between the beginning and end of the wave.

All slow waves were sorted into 5 amplitude percentage ranges (5 quintiles, 20 percentiles each) according to the amplitude of the peak (from zero-crossing to maximum value), as it was shown in previous studies^18^,^19^ and all the parameters of individual slow waves were computed for each amplitude percentage range, since, as it is reported previously, the most important determinant of power in the SWA band is the number of high-amplitude slow oscillations^20^. The above-mentioned analysis was performed in the 6 months old (n = 9) and 18 months old (n = 9) group.

## Results

### Vigilance states and episode duration

**Figure 1:**
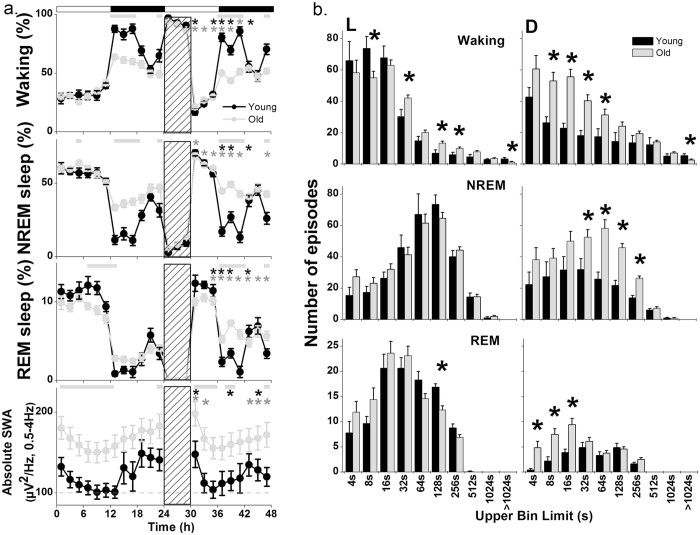
Time course of vigilance states and absolute slow-wave activity (SWA, 0.5–4* *Hz) in non-rapid-eye movement (NREM) sleep, and episode frequency histograms of vigilance states. (**a**) Time course of vigilance states and absolute SWA in NREM sleep, for 24-h baseline, 6-h sleep deprivation (SD, hatched bar) and 18-h recovery for the two age groups, young (black circles, n = 9) and old mice (gray circles, n = 24). Curves connect 2-h values (mean ± SEM) of Waking, NREM, REM sleep and absolute SWA. The black and white bars indicate the light-dark cycle. Horizontal gray lines represent significant differences between young and old across the 48-h period and black (young) and gray (old) asterisks significant differences between recovery and baseline day (unpaired and paired t-tests, p < 0.05 after significant 1-, 2- or 3-way ANOVA, interaction factors ‘age’, ‘age*time of day’ and ‘age*time of day*day’). (**b**) Episode frequency histograms of Waking (upper), NREM (middle) and REM sleep (lower panel) in the Light (L, left) and Dark (D, right) period during baseline for young (black bars, n = 9) and old mice (gray bars, n = 24) (mean values ± SEM). Episodes are partitioned into ten exponentially increased duration bins from 4 to >1024 s (x axis designates the upper limit of each bin for the following bins: 0–4, 5–8, 9–16, 17–32, 33–64, 65–128, 129–256, 257–512, 513–1024, >1024). Asterisks indicate significant differences between young and old animals (unpaired t-tests, p < 0.05 after significant 2- or 3-way ANOVA, interaction factors ‘age*duration’, ‘age*Light-dark’ and ‘age*Light-dark*duration’).

The aged mice showed increased NREM sleep and decreased waking, especially in the dark period (Fig. 1a) (2-way ANOVA, interaction factors ‘age*time of day’; Waking: p < 0.0001, NREM: p < 0.0001). During undisturbed BL, REM sleep was decreased in the older animals at the end of the light period and increased during the first part of the dark period, as compared to the young animals. Also in the dark period after SD, REM sleep was increased in older mice as compared to young controls (2-way or 3-way ANOVA, interaction factors ‘age*time of day’; REM sleep: p < 0.0001 and ‘age*time of day*day’; REM sleep: p < 0.0001).

Episodes of each vigilance state were partitioned into ten bins with exponential increased duration from 4 s to >1024 s^34^,^35^,^36^ (Fig. 1b). The largest differences between young and old animals were found in the dark period. During both dark periods, aged animals showed increased number of the longer NREM sleep episodes (2-way or 3-way ANOVA, interaction factors ‘age*duration’; p = 0.016, ‘age*Light-dark’; p < 0.0001, ‘age*Light-dark*duration’; p = 0.053), more short and middle duration waking episodes and a decrease in the longest waking episodes (>1024 sec) compared to young controls. In the BL light period, older animals had fewer short waking episodes and a higher incidence of longer episodes (2-way or 3- way ANOVA, interaction factors ‘age*duration’; p = 0.002, ‘age*Light-dark’; p < 0.0001, ‘age*Light-dark*duration’; p = 0.001). In both light periods, older mice showed a decrease in the 128 s-bin REM sleep episodes and in the dark period an increase in the number of shorter REM sleep episodes compared to the young animals (2-way or 3- way ANOVA, interaction factors ‘age*duration’; p < 0.0001, ‘age*Light-dark’; p < 0.0001, ‘age*Light-dark*duration’; p = 0.730) (data shown only in BL).

### Absolute EEG power density and absolute SWA

**Figure 2:**
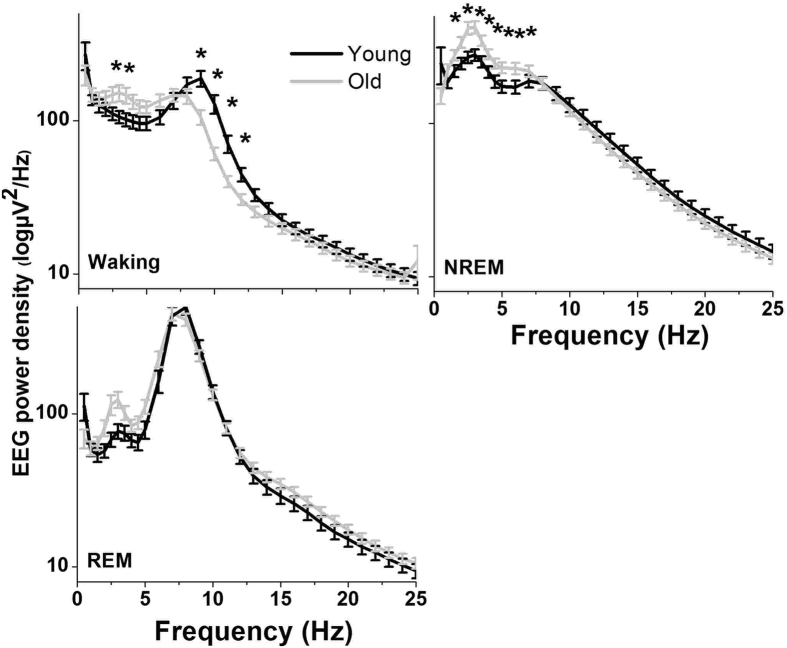
Spectral distribution of electroencephalogram (EEG) power density (mean values ± SEM) in the frequencies between 0.5 and 25 Hz for young (black, n = 9) and old mice (gray, n = 24) in Waking (top left), non-rapid-eye movement (NREM, top right) and REM sleep (bottom left) computed for pooled values of the 24-h baseline day. Asterisks indicate significant differences across the frequency bins between the two groups (unpaired t-tests, p < 0.05 after significant 2-way ANOVA, interaction factors ‘age*frequency bins’).

To investigate whether gross alterations in sleep-wake amount and distribution are associated with specific changes in cortical EEG, we calculated the spectral distribution of absolute EEG power density in the frequencies between 0.5 and 25 Hz (Fig. 2). Aged mice had a significantly higher EEG power density in the frequencies between 2–7 Hz during NREM sleep, as compared to young controls. In waking, EEG spectra in old animals were characterized by a pronounced decrease in power density on the high end of the theta peak (9–12 Hz), whereas power density around 3 Hz was slightly higher. These observed differences were not only frequency- but also vigilance state specific, as no significant differences were found in the REM sleep EEG spectra (2-way ANOVA, interaction factors ‘age*frequency bins’; Waking: p < 0.0001, NREM sleep: p < 0.0001, REM sleep: p = 0.332).

In the old mice, higher absolute SWA in NREM sleep was observed over the entire 48 h recording period (one-way ANOVA, factor ‘age’; p < 0.0001, (t, df) =(9.329, 46); p < 0.0001) with significant differences during the entire light period and at the end of the dark period, where most sleep occurs (Fig. 1a, bottom panel). Further age differences were apparent in the dynamics of SWA after sleep deprivation. Specifically, the decay rate in NREM sleep SWA between the first and third hour after SD, was significantly slower in the old animals [young: 154.58% ± 7.55; old: 138.04% ± 3.75, unpaired t-test (t, df) =(2.164,31); p = 0.038, Supplementary material, Fig. S1].

### Slow-wave morphology

#### Average EEG slow wave slopes and sigma activity

**Figure 3:**
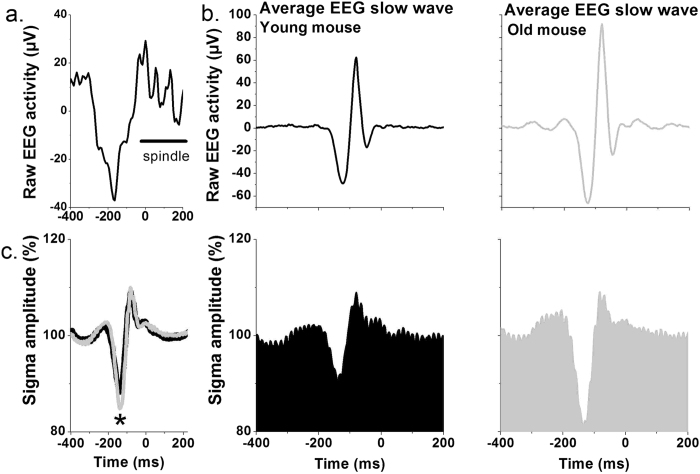
Average electroencephalogram (EEG) slow wave and Sigma amplitude. (**a**) Representative EEG trace of a slow wave followed by a spindle. Time point of 0 ms was arbitrarily defined to emphasize the example of the spindle-event that follows a random slow-wave. (**b**) Representative average EEG slow wave (μV, top graphs) and Sigma amplitude (%, 9–13 Hz, bottom graphs) of one young (left, black) and one old mouse (right, gray). (**c**) Average Sigma amplitude (%, mean values ± SEM) in the young (black, n = 9) and aged mice (gray, n = 9). The asterisk indicates significant difference between young and old mice for the period of the trough (unpaired t-test, p = 0.0286).

An example of a trace of raw EEG from a young mouse, with a slow wave followed by a spindle-event, defined as burst of activity in the range between approximately 9–13 Hz, is shown in Fig. 3a. In Fig. 3b, two examples are depicted, which show the average EEG slow wave from an individual young and an old mouse, along with the corresponding rectified amplitude of the EEG signal, filtered in the sigma frequency range (9–13 Hz). Notably, a higher amplitude slow wave is observed in the older animal. In the average values, steeper absolute first and second slopes were found in the old compared to young mice, in the main sleep period [1^st^ slope, Young: 808.01 μV/s ± 8.61; old: 1018.1 μV/s ± 8.44, unpaired t-test (t, df) =(17.429, 538); p < 0.0001, 2^nd^ slope, Young: 941.15 μV/s ± 8.82; old: 1126.8 μV/s ± 6.94, unpaired t-test (t, df) =(16.541, 509.9); p < 0.0001], and increased absolute slow-wave amplitude and slopes in the old compared to the young, whereas no significant differences were found in the slow-wave duration (Fig. S2, data of the highest-amplitude slow waves shown). Furthermore, we observed a deeper trough in the average sigma activity, corresponding to the negative phase of the slow wave, in the old as compared to the young mice [Fig. 3c, unpaired t-test for the trough period (t, df) =(2.252, 52); p = 0.0286].

#### Slow-wave Incidence and Multipeak Waves

**Figure 4:**
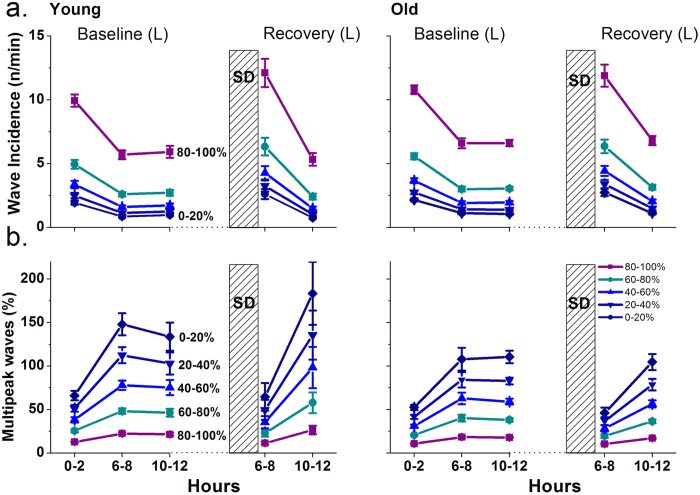
Slow-wave incidence (n/min) and number of slow waves with more than one peak (multipeak waves) during five 2-h time points across the light period of baseline and recovery for young (left, n = 9) and old mice (right, n = 9). Slow waves were equally subdivided into five amplitude quintiles ranging from 0–100%. (**a**) The wave incidence (mean values ± SEM) changes significantly in the following time points in all amplitude quintiles in both groups: 0–2 h vs. 10–12 h in baseline, 6–8 h vs. 10–12 h in recovery, 6–8 h in baseline vs. 6–8 h in recovery (paired t-tests, p < 0.05 after significant 2-way ANOVA, interaction factors ‘time of day*amplitude levels’). (**b**) Multipeak waves are expressed as a percentage of the total number of slow waves (mean values ± SEM). They change significantly in the following time points in all amplitude quintiles in both groups: 0–2 h vs. 10–12 h in baseline, 6–8 h vs. 10–12 h in recovery, 6–8 h in baseline vs. 6–8 h in recovery. Significant differences were found between the groups, with old mice having overall less multipeak waves (paired and unpaired t-tests, p < 0.05 after significant 2-way ANOVA with interaction factors ‘age*time of day’ and ‘age*amplitude levels’).

To investigate the origin of the differences in absolute SWA between young and old animals, we analyzed the incidence of individual slow waves, in five 2 h values, during the BL sleep period and during sleep after SD. To this end, all slow waves were sub-divided into five amplitude quintiles ranging from 0–100%, as described previously^18^,^19^, within three time points during BL (0–2, 6–8, 10–12 h) and two time points during sleep after SD (6–8, 10–12 h) (Fig. 4a). No differences in the incidence of slow waves were found between the age groups. In both age groups, slow-wave incidence significantly decreased across the BL sleep period and significantly increased after SD, and this was observed for slow waves within each 20% quintile (2-way ANOVA, interaction factors ‘time of day*amplitude levels’; p < 0.0001, ‘age*time of day’; p = 0.275, ‘age*amplitude levels’; p = 0.428). Similar significant decreases were found between the first and last 2 h values of the 6-h interval after SD.

In both groups, the number of multipeak waves (number of peaks within a single wave, five 2 h values expressed as percentage of the total number of waves during each time point at the respective amplitude quintile) was lowest among the highest-amplitude slow waves, but invariably an increase in the course of the main sleep period was observed (Fig. 4b). An age-dependent difference was apparent, manifested in significantly lower proportion of multipeak waves in older animals, as compared to young controls (Fig. 4b, 2-way ANOVA, interaction factors ‘age*time of day’; p = 0.001, ‘age*amplitude levels’; p = 0.003). On average, in the aged mice 1.14 peaks per slow wave occurred, in the main sleep period, whereas, young controls had 1.22 peaks per slow wave (unpaired t-test (t, df) =(-5.491, 55.241); p < 0.0001).

**Figure 5:**
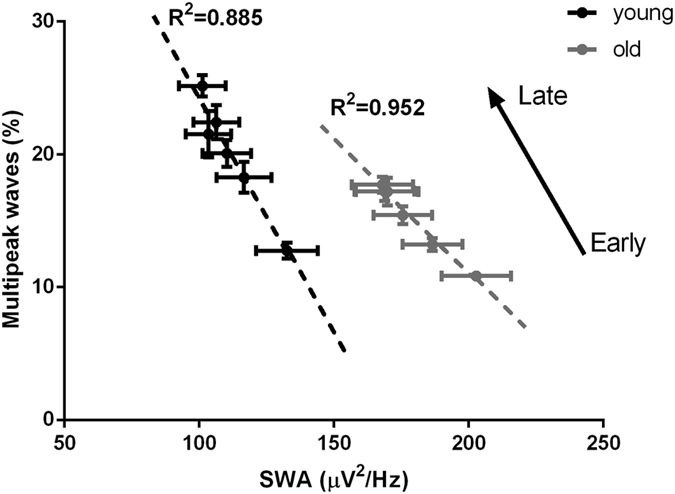
Relationship between absolute non-rapid-eye movement (NREM) sleep slow-wave activity (SWA, 0.5–4 Hz) and multipeak waves (expressed as a percentage of the total number of slow waves) computed for the six 2-h intervals across the light period (time points 0–2, 2–4, 4–6, 6–8, 8–10, 10–12 h) of baseline for young (black, n = 9) and old mice (gray, n = 9) for the highest-amplitude slow waves (80–100%) (mean values ± SEM). Dashed lines depict linear regression (R^2^ are significantly different between young and old mice, unpaired t-test p = 0.013).

Previously, a negative correlation was found between the number of multipeak waves and SWA in young rats and humans^18^,^19^. Since both variables showed significant changes in the course of aging, we conducted a regression analysis, to determine whether the relationship between multipeak waves and absolute SWA was influenced by aging. In both groups, 2 h values obtained during the BL light period (six time points at 0–2, 2–4, 4–6, 6–8, 8–10, 10–12 h) of the highest-amplitude multipeak waves (expressed as percentage of the total number of waves during each time point, at the 80–100 amplitude quintile) were significantly negatively correlated with the six corresponding 2 h values of absolute SWA, obtained during the BL light period. (R^2^ in young: 0.885; old: 0.952, Fig. 5). However, the age groups formed two distinct clusters where the slope (young: −0.3921 ± 0.0512; old: −0.2231 ± 0.0179; p = 0.007), and intercept (young: 60.807 ± 3.005; old: 54.67 ± 2.84; p = 0.034) of the regressions differed significantly.

### Slow-wave slopes

**Figure 6:**
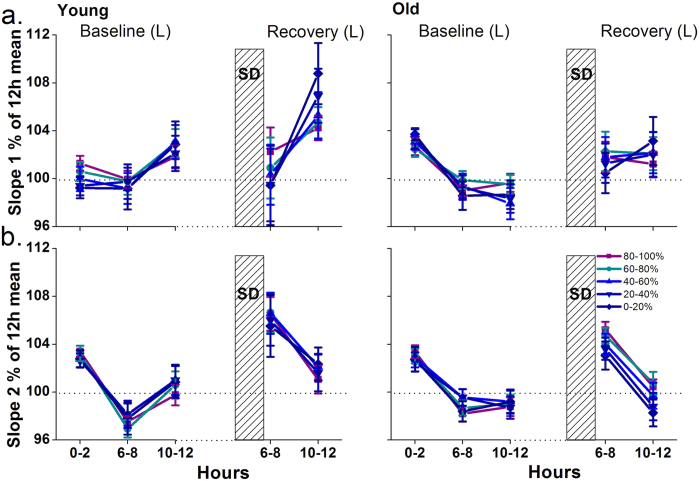
Slope of the first and second segment of the slow waves during five 2-h time points across the light period of baseline and recovery for young (left, n = 9) and old mice (right, n = 9). Slow waves were equally subdivided into five amplitude quintiles ranging from 0–100% and slopes (mean values ± SEM) are expressed as % of the 12 h mean. (**a**) The slope of the first segment (Slope 1) changes significantly in the 0–2 h vs. 10–12 h in baseline, only in the old mice in all amplitude quintiles (paired t-tests, p < 0.05 after significant 2-way ANOVA with interaction factors ‘age*time of day’). (**b**) The slope of the second segment (Slope 2) changes significantly in the following conditions: 0–2 h vs. 10–12 h in baseline in young mice in the 80–100% quintile, 0–2 h vs. 10–12 h in baseline in old mice in all amplitude quintiles, 6–8 h vs. 10–12 h in recovery in the young mice in the 80–100% quintile, 6–8 h vs. 10–12 h in recovery in the old mice in all amplitude quintiles, 0–2 h in baseline vs. 6–8 h in recovery in the old in the 80–100% quintile and 6–8 h in baseline vs. 6–8 h in recovery in all amplitude quintiles in the young and old mice (paired t-tests, p < 0.05 after significant 2-way ANOVA with interaction factors ‘age*time of day’).

Age-dependent differences in multipeak slow waves suggest that network synchronization may be altered in older animals. Therefore, next, we investigated the dynamics of changes in the slopes of the slow waves, so that conclusions can be drawn in the comparison with older humans^5^,^6^ as well as young rats^18^. The slope of the first and the second segment of the slow wave was computed in five 2 h values across the BL sleep period (0–2, 6–8, 10–12 h) and after SD (6–8, 10–12 h) for the five amplitude quintiles (Fig. 6). The slope of the first segment (Slope 1, expressed as % of the 12 h mean) did not show noticeable changes, in the course of the main sleep period, in the young mice, whereas, significantly steeper slopes were observed during early sleep in the old mice (Fig. 6, 2-way ANOVA, interaction factors ‘age*time of day’; p < 0.0001). After SD, no significant change in the 1^st^ slope was found in either group.

The second segment of the slow-wave slope (Slope 2, expressed as % of the 12 h mean) changed in the course of the BL sleep period in both age groups, however with a different time course (2-way ANOVA, interaction factors ‘age*time of day’; p = 0.001) (Fig. 6b). Steeper 2^nd^ slopes were observed in the first 2 h of the main sleep period in the highest-amplitude slow waves in the young (80–100% quintile) whereas in the older animals significant changes were found in all slow waves, irrespective of amplitude. After SD, steeper 2^nd^ slopes were found only among the highest-amplitude slow waves in the young mice, whereas in the older mice slow-wave slopes were initially higher and subsequently declined, irrespective of slow-wave amplitude. No significant changes in slopes were obtained for the first 2 h in BL vs. the first 2 h of the recovery condition for the young mice, but steeper slopes were apparent for the highest-amplitude slow waves in the older mice. For the 6–8^th^ h in BL vs. corresponding time after SD, significant steeper slopes were obtained after SD, in all amplitude quintiles, in both age groups.

Overall, these findings reveal an effect of age on slow-wave slopes. Within the older group, we found a consistent generalized decrease in slopes in the course of the main sleep period among both low- and high-amplitude slow waves. In contrast, in the young mice, only the second slope of the high-amplitude slow waves (80–100% quintile) decreased significantly in the course of the day, whereas it was not the case for the slope of the lower-amplitude slow waves or the first slope.

## Discussion

We conducted EEG and EMG recordings and performed a detailed analysis of individual EEG slow waves in healthy young and older mice, and show that older mice display significant changes in a broad range of sleep and EEG parameters, compared to young mice. Many of the changes suggest persistently elevated sleep pressure in the older mice. Similar to the effects after SD^33^,^36^,^45^,^46^, older mice slept more and had longer NREM sleep episodes during the dark period. Furthermore, older animals exhibited increased EEG power density during NREM sleep in slow frequencies (2–7 Hz) and, compared to young controls, had an overall higher level of NREM sleep SWA, which is also typical for increased sleep pressure^47^,^48^,^49^. Consistently, analysis of slow-wave characteristics revealed that older mice had fewer multipeak waves, steeper absolute slow-wave slopes and more pronounced changes in the relative slow-wave slopes, similar to the effects of sleep deprivation^18^. Interestingly, the modulation of sigma amplitude in association with the occurrence of individual slow waves was also stronger in older animals, suggesting a tighter synchronization among cortical neuronal populations during both the network activity and silence. This is in line with increased sleep pressure, as it was previously shown, that network synchronization increases during sleep, under elevated sleep pressure^40^. Finally, the decay rate of SWA after SD was reduced in older mice, which is indicative of a slower recovery process. Altogether, the data suggest that brain network properties may be altered in older mice. We hypothesize that these changes result in a less restorative sleep, which prevents efficient dissipation of sleep pressure, and leads to a chronically sleep deprived state.

### Sleep architecture is affected by aging

Older mice had increased NREM sleep in the dark period, and reduced REM sleep at the end of the light phase. These findings are in accordance with previous findings in older mice^7^,^8^,^9^,^10^,^11^,^12^. For example, previous work in C57BL/6J mice, where 23 months old mice were compared to 3 months old^7^ or 21 months old mice to 5 months old^9^ or 22–24 months old to 2–4 months old^10^ revealed increased NREM sleep in the Dark period and decreased REM sleep in the light. In another study^8^, an increase in NREM and REM sleep from the age of 3 months to 1 year in DBA/2J, C57BL/6J mouse strains, and in NREM sleep in AKR/J mice, was reported, whereas at the age of 24 months old a decrease in NREM was reported in the C57BL/6J and DBA/2J mice as compared to the 1 year old, and a decrease in REM sleep in the C57BL/6J mice as compared to the 1 year old and 6 months old mice and in the DBA/2J mice as compared to the 1 year old mice. Some studies, therefore, found a decrease in sleep in 2 year old mice compared to 1 year old mice, but most found a steady increase in sleep in the course of aging. None of the mouse strains at higher age (>18 months old) showed a decrease in sleep below the level of 3 months old. In general, we can conclude that our findings are in accordance with most studies regarding the C57BL/6J strain, but similar changes in sleep architecture were also found in other mouse strains.

Furthermore, we found in the older animals more long NREM sleep episodes and more short wake and REM sleep episodes during the dark phase compared to the young animals, similar to a previous study^10^.

Sleep deprivation did not influence the differences in waking and NREM sleep between young and older mice, however, older mice showed a clear increase in REM sleep compared to BL in the dark period which was not observed in the young group.

The physiological mechanisms underlying age-induced changes in sleep architecture have yet to be determined. It was proposed that alterations in neurotransmitter and receptor balance, such as increased adenosine, a ‘sleep facilitator’ and decreased hypocretin (orexin), a ‘wakefulness regulator’, as well as alterations in the glutamatergic and GABAergic systems, can lead to sleep-wake dysregulation^50^,^51^,^52^,^53^. Moreover, changes in the output strength of the circadian clock may underlie the changes seen in the course of aging^54^. In that context, the increase in the amount of sleep during the dark phase may be caused by lower circadian amplitude, causing a weaker waking signal coming from the SCN in the active period of the mice.

### Aging is associated with changes in EEG power density

In contrast to humans^1^,^2^,^3^,^5^,^6^,^37^,^38^, the EEG in older mice was characterized by higher absolute power density in the EEG slow wave range, as compared to young mice. In the waking spectrum, less power density was found in the 5–12 Hz frequency range and more power density in the lower frequencies around 3 Hz in the older mice. These findings were specific for the respective vigilance state and no differences were observed in the REM sleep spectrum. They are, therefore, not caused by a general unspecific change in the EEG activity of older mice, suggesting that NREM sleep EEG regulatory mechanisms are likely to be more affected compared to REM sleep in aging.

The difference in 5–12 Hz activity during waking can be explained by differences in the amount of active waking. In rodents, theta activity in the EEG is increased during active wakefulness, for example during exploration, compared to quiet waking^55^,^56^. The lower theta activity in the older mice is in accordance with previous findings, showing possibly less exploratory behavior and reduced arousal in older compared to young mice^10^. Furthermore, the increase in EEG spectral power around 3 Hz in waking is reminiscent of the effects of sleep deprivation, and may reflect intrusions of sleep-like network activity into the awake state^21^. This, also, agrees with the notion that sleep pressure in older mice is elevated.

Older animals had an overall elevated level of SWA in the NREM sleep EEG (Fig. 1a). NREM sleep SWA is the best characterized marker of sleep intensity and increases in response to sleep loss^16^. It decreases in the course of the main sleep period and gradually increases in the dark period, when the animals are more awake and active, a pattern visible in both groups. However, older mice showed higher absolute SWA values across the whole recording period, with significant differences compared to the young animals, particularly in the light period and at the end of the dark period. These observations contrast human studies, where absolute SWA values, as well as absolute amplitude, frequency and density of slow waves are found to be decreased in aging^1^,^2^,^3^,^5^,^6^,^37^,^38^.Moreover, the decay rate in SWA in the first three hours after SD was slower in the older mice compared to the young controls. The latter suggests that sleep in older mice is less efficient in dissipating sleep pressure. Previous studies in mice suggested that absolute levels of SWA correlate with homeostatic sleep pressure^47^,^48^,^49^. Therefore, the higher level of absolute SWA in our data supports the notion of an increased sleep pressure phenotype in older mice.

### Age-dependent changes in SWA arise from changes in slow-wave morphology

To better understand the network mechanisms, underlying the differences in SWA between young and older mice, we assessed several characteristics of individual EEG slow waves, as it has been performed in earlier studies in the rat^18^ and in humans^5^,^6^,^19^. The dynamics of slopes of the slow waves in old mice showed similar properties to the study conducted in the rat^18^. In young mice only the 2^nd^ slope of the high-amplitude slow waves (80–100% quintile) changed significantly in the course of the day, whereas in older mice changes were found in all slow wave amplitude ranges both in the 1^st^ and 2^nd^ slopes. The time course of the changes in these slopes showed similar dynamics as the time course of SWA. Increases in the 2^nd^ slope were also found after sleep deprivation and this is therefore an indication of increased sleep pressure in the old mice^18^. The absence of changes in the 1^st^ slope, in the course of the main sleep period in the young mice, suggests a lower network synchronization into the silent state of the neurons in young mice, compared to the older mice. In middle-aged (41–60 y.o.)^5^ and older humans (50–70 y.o.)^6^, an age-related decrease in absolute slow-wave slope was found, in contrast to our data in mice.

The cellular counterpart of EEG slow waves - the slow oscillation - consists of an up state, when neurons are depolarized, and a down state, when neurons are hyperpolarized and spiking is diminished. Overall, NREM sleep SWA is a reflection of near-synchronous transitions between up and down states among the large population of cortical neurons near the recording electrode^39^,^40^,^43^. Evidence suggests that the more synchronous the firing pattern of single neurons is, the higher the amplitude of corresponding EEG slow waves, and the steeper the slow-wave slopes. Therefore, slow-wave slopes can be considered as indicators of neuronal synchronization and, indirectly, the synaptic strength in the cortex^18^,^20^. Moreover, changes in the slow wave parameters are mediated by changes in the amplitude of single-cell oscillations, in the dynamics of network synchronization, and in the rate of neuronal recruitment and decruitment^20^,^28^. Therefore, the increased absolute SWA and slow-wave slopes, the decreased sigma activity during the down state, and the decrease in multipeak waves that we found, possibly indicate that more neurons are recruited in producing the overall slow wave in the older mice, suggesting increased neuronal coupling at the local level between cortical neurons in this group.

Consistent with previous studies^18^,^19^, in our data the incidence of slow waves is positively correlated with absolute SWA whereas multipeak waves follow an opposite pattern. Notably, older animals had fewer multipeak waves across the day compared to young controls. Multipeak waves reflect the activity of asynchronous spatially distinct clusters of neurons due to poor spatial synchronization when the overall connection strength is reduced^20^. In both age groups, the number of multipeak waves increased in the course of prolonged sleep, similar to previous data obtained in rats and humans^18^,^19^. However, when analyzing the relationship between SWA and multipeak waves, the age groups separated into two clusters. Multipeak waves are independent of amplitude and, hence, this shows that our findings are not merely based on an amplitude difference between the two age groups, but that the morphology of the slow waves is indeed altered between the age groups. This, together with the difference found in sigma activity during the down state of the slow wave, suggests a consistent change in cortical network connectivity, which possibly develops in the course of aging. In young mice, high global synchronization probably occurs together with possibly relatively lower local one, ending up in an increased number of up states and down states that effectively cancel each other out. At the cortical level, the relative synchronization observed during slow-wave sleep is likely the result of the high interconnectivity of the thalamocortical system^20^. Taking into account that age-related sleep impairments are unlikely to be due to neuronal loss, but due to changes in synaptic connectivity^57^ and, thus, loss in neuronal connections, our data suggest neuronal connectivity changes in the cortical network, in the course of aging, possibly towards weaker global and stronger local synchronization. However, as we did not measure unit activity of neurons, more research is needed to investigate this further.

Recent research, applying *in-vivo* two-photon imaging that tracked the size and location of axonal boutons in aged mice, revealed an increase in synaptic structural dynamics in specific cortical circuits in the aged cortex^58^. Increased rates of synapse formation, elimination and destabilization, and decreased synaptic tenacity in the aged cortex could potentially cause the slow wave alterations that we detect through EEG recordings reflecting the increased network connectivity in local circuits.

Furthermore, it has been shown in aged mice that mean postsynaptic density synapse length is greater and that there is selective maintenance of large prefrontal spines with reduced plasticity^59^. Both aforementioned studies suggest alterations in the aging cortex regarding the synapse and the structural connectivity and plasticity that may lead to the increased network connectivity in local circuits suggested by our results.

### From mice to humans

Although at a first glance the changes in cortical network connectivity in the course of aging seem to differ between humans and mice, further research, from large-scale electric signals to single-cell recordings, is undoubtedly necessary. The EEG electrodes used in mice and humans differ significantly in size, and so does the size of the neuronal population recorded. It is possible that intracerebral recordings in humans, where a small network contributes to the field potential picked up by the electrode, may reveal similar differences to those found in mice. Volume conduction may have a considerably greater influence on the EEG waveforms in humans. The possibility remains that long-distance connectivity is reduced, both in humans and mice, but this occurs on a spatial scale, which cannot be compared directly. In an earlier study in humans (3 age groups: 20–30 y.o, 31–45 y.o., 45–60 y.o.)^60^, an increase in EEG dimensional complexity was revealed with age, supporting the hypothesis that the complexity of brain dynamics increases with brain maturation. It was argued that this may be caused by an increase in the number of functionally related cell assemblies oscillating at their own frequencies. This may be the equivalent to the increase in slope and amplitude found in NREM sleep slow-waves in the older mice.

Furthermore, dysfunctional circadian rhythms potentially related to sleep-wake disturbances have been reported as an explanation of the changes found in sleep as humans age. In our results, the most profound differences between the age groups were found in the dark phase of the day, the active period of nocturnal animals, which is in accordance with previous reports^7^,^8^,^9^,^10^,^11^,^12^. With age, NREM sleep, and especially the longer episodes, increased during the night. This could be equivalent to the increased daytime sleepiness and decreased sleep at night described in elderly humans^61^,^62^,^63^. These changes in sleep architecture in humans and mice may be related to a decrease in the amplitude of the circadian clock^54^. Since SCN neuronal activity is similar in diurnal and nocturnal animals, this results in increased sleep in the active period in mice and decreased sleep in the rest period in humans.

Contrary to human data 1,2,3,5,6,37,38, aged mice demonstrated an increment in the absolute SWA, amplitude and slow-wave slopes. The aged mice in our study were between 18–24 months old, which is approximately equivalent to 50–70 years in humans. The human subjects in the studies that investigated the slow-wave characteristics, in parallel with the SWA, were middle aged (41–60 years of age)^3^,^5^ or older (50–70)^6^. It is, therefore, unlikely that the differences between humans and mice are caused by an inappropriate age match. Therefore, in the course of aging, the cortical network seems to undergo changes which are reflected in the NREM sleep EEG. These EEG changes support the notion of a higher sleep pressure in the aged mice, contrasting what has been found in the elderly humans. This, does not render the mouse model, specifically the C57BL/6J strain, invalid for sleep and aging research, however, these specific differences have to be taken into account when comparing between species.

## Additional Information

**How to cite this article**: Panagiotou, M. *et al*. Differences in electroencephalographic non-rapid-eye movement sleep slow-wave characteristics between young and old mice. *Sci. Rep.* **7**, 43656; doi: 10.1038/srep43656 (2017).

**Publisher's note:** Springer Nature remains neutral with regard to jurisdictional claims in published maps and institutional affiliations.

## Supplementary Material

Supplementary Information
