# Supplementary material for: Differences in electroencephalographic non-rapid-eye movement sleep slow-wave characteristics between young and old mice
Source: Sci Rep. 2017 Mar 3;7:43656. doi: 10.1038/srep43656 (PMC5334640; doi:10.1038/srep43656)
Supplement: Supplementary Information [file srep43656-s1.pdf]

Differences in electroencephalographic non-rapid-eye movement sleep slow-wave characteristics between young and old mice

Maria Panagiotou[a], Vladyslav V. Vyazovskiy[b], Johanna H. Meijer[a], Tom Deboer[a,*]

[a]Laboratory for Neurophysiology, Department of Molecular Cell Biology, Leiden University Medical Centre, 2333 ZC Leiden, The Netherlands [b]Department of Physiology, Anatomy and Genetics, University of Oxford, OX1 3PT Oxford, UK

[*]To whom correspondence should be addressed. Email: tom.de_boer@lumc.nl

Supplementary material

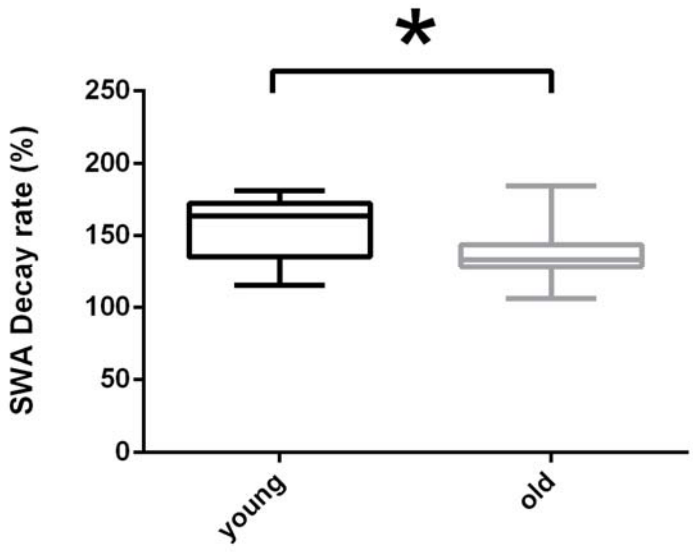

Fig. S1: Decay rate of slow-wave activity (SWA) for the first three hours after sleep deprivation (SD) for young (black, n=9) and old mice (gray, n=24). The asterisk indicates significant difference between the two age groups (unpaired t-test, p=0.038).

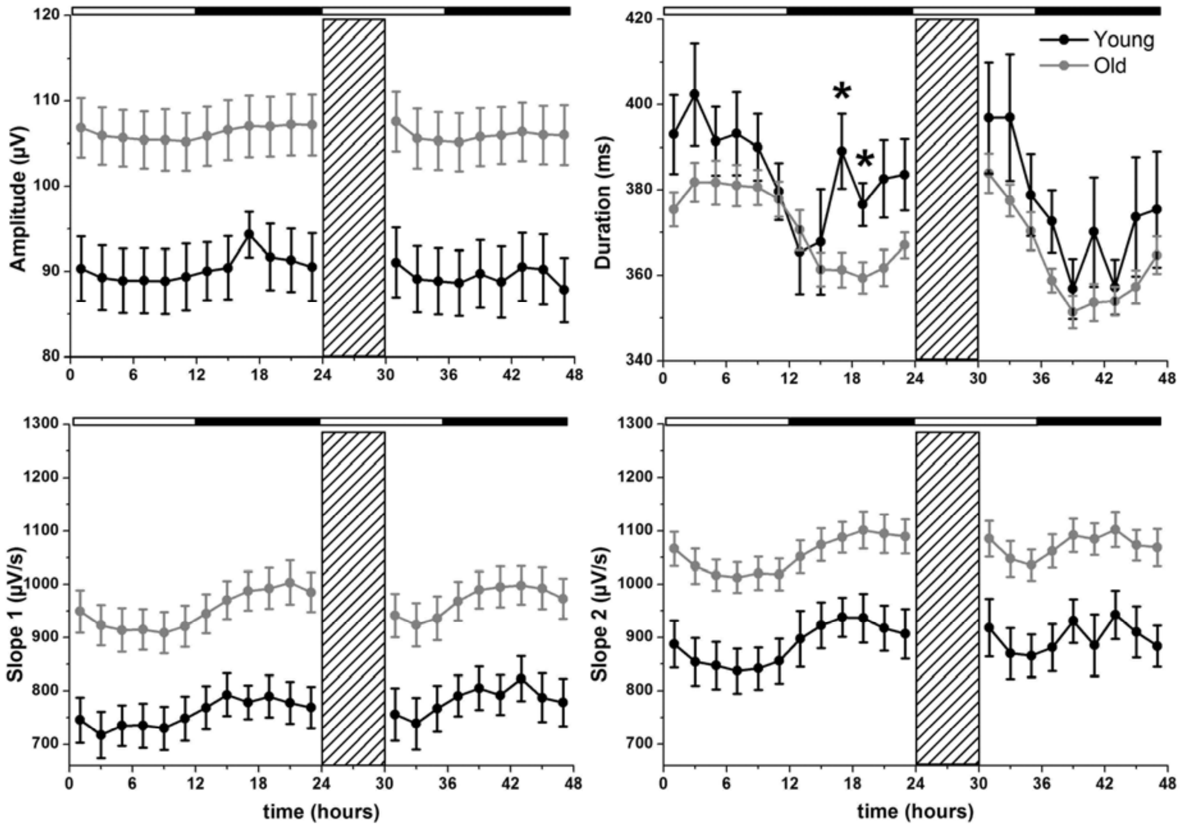

Fig. S2: Time course of absolute slow wave amplitude, duration, first and second slope of the highest-amplitude (80-100%) slow waves for 24-h baseline, 6-h sleep deprivation (SD, hatched bar) and 18-h recovery for the two age groups, young (black circles, n=9) and old mice (gray circles, n=9). Curves connect 2-h values (mean ± SEM), black and white bars indicate the light-dark cycle and asterisks indicate significant differences between young and old animals (unpaired t-tests, $p<0.05$ after significant ANOVA). For the amplitude, duration and slopes higher absolute values were observed over the entire 48h recording period (one-way ANOVA, factor 'age'; $p<0.0001$).
